# Conversations on death and dying: exploring performance as a prompt

**DOI:** 10.1177/26323524231209059

**Published:** 2023-11-01

**Authors:** Sheila McCormick

**Affiliations:** School of Arts, Media and Creative Technology, The University of Salford, Room 5.12, New Adelphi Building, Salford M5 4BR, UK

**Keywords:** autobiography, death, dying, palliative care, performance

## Abstract

**Background:**

Death is inevitable, yet for some, conversations around death remain difficult. The stigmatisation of death amongst some cultures has a negative impact with studies showing societies least likely to discuss end of life openly remain the lowest ranked in terms of end-of-life care quality. Out of this understanding have come several socially engaged projects (e.g. Death Cafes, The Conversation Project, Before I Die Festivals) developed to encourage engagement with the subject.

**Objective:**

In this article I ask, can autobiographical performance prompt conversations on death and dying? To answer the research question, I examine the socially engaged Death, Dinner, and Performance project, and analyse the effectiveness of the performance/dramaturgical methodology developed in the project to encourage participant engagement with the difficult subjects of death and dying.

**Design:**

I look specifically at the use of autobiographical performance strategies in the Death, Dinner, and Performance project and explore the outcomes associated with the adaptation of those strategies (particularly regarding relationality in a socially engaged context) in conversations between participants on death, dying and bereavement.

**Method:**

The project adopted a mixed methodology that engaged both Practice as Research (PaR) and qualitative research strategies.

**Results:**

PaR reflection and analysis, along with qualitative coding of participant responses allowed an inductive, thematic analysis that highlighted several recurring themes. These are analysed and discussed under two categories in the Analysis and results section at the end of this paper: firstly, in relation to recurring themes in the participants’ discussion around the subject of death and dying, and secondly, in relation to the socially engaged strategy (commensality and use of autobiographical performance) used to encourage that discussion.

## Introduction

In this article I ask, can autobiographical performance provide a prompt for conversations on death and dying? To do so, I explore the outcomes of a Practice as Research (PaR) project entitled Death, Dinner, and Performance: A Study of the Efficacy of Performance to Enhance Conversations Around Death and Dying. The project brought together commensal practices and autobiographical performance strategies to explore the development of a performance/participation method used to encourage conversation around the subjects of death and dying.

As well as addressing the initial research question: can autobiographical performance provide a prompt for conversations on death and dying?, the PaR also allowed the following to be explored: the efficacy of the performance material to prompt conversation around death and dying; the efficacy of the theatrical strategies to encourage those conversations to develop; and the efficacy of the event to encourage ongoing reflection in participants on death and dying and conversations therefrom.

### Positionality statement

The project developed from earlier research on ageing and creative applied practice that culminated in the publication of my monograph *Applied Theatre: Creative Ageing* (Bloomsbury, 2017). The Death, Dinner, and Performance project was also informed by my previous professional experience as a Registered General Nurse working in palliative and end-of-life care.

### Background

There is a range of misconceptions that surround dying, death and bereavement.^1–3^ Because of this, there is a growing consensus that palliative care needs to encompass a health-promoting element to encourage openness about death which, in turn, will inspire people to develop ways to live and support each other with death, dying and bereavement.^4,5^ Research shows that a lack of communication about end-of-life preferences is one of the main reasons people do not receive the care they prefer, which is often palliative rather than interventional.^6^ Thus, avoiding discussions on end-of-life results in greater healthcare spending and more unwanted hospital admissions.^7^ Considering this, a review of applied theatre and socially engaged projects^8^ showed little work (particularly in terms of dialectical performance) in the areas of death and dying.

### Purpose

In his book *Theatre Death*, Robson suggests, ‘If death is not to dominate us, we have to learn to live with it. This must have a double focus: how to live with the shadow of our own mortality, and how to survive the death of others’^9^ (p. IX). One of the aims of the Death, Dinner, and Performance project was to consider whether a performance methodology could be developed to encourage open discussion around death and to consider what Robson suggests is, ‘theatre’s persistent confrontation with death [. . .as] one of the vital ways in which it continues to find an ethical and political force’^9^ (p. IX).

Death remains taboo^10^ in certain contexts and this taboo can have a negative impact with studies showing the most death-adverse countries (and thus those least likely to discuss openly end of life) remain the lowest ranked in terms of end-of-life care quality.^6^ Out of this understanding have come several successful initiatives developed to encourage engagement with this difficult subject including Death Cafes, Death Over Dinner, The Conversation Project and Before I Die Festivals. The Conversation Project, for example, is a public engagement initiative supported by the Institute for Healthcare Improvement. According to its website, more than 600,000 people from 160 countries download its Conversation Started Guide in multiple languages.^11^ At its core, the Death, Dinner, and Performance project builds on these initiatives.

In this article, I explore a performance strategy (autobiographical performance) and ritualised, commensal event (death dinner). I do so to question the efficacy of intimate, autobiographical performance in a communal, commensal setting to transgress taboo and enable access to the difficult subjects of death, dying and bereavement. In doing so, I provide new knowledge and understanding of how performance and performance strategies can help to address the need for more open and engaged conversations around death, dying and end-of-life preferences.

### Participants

As this was a pilot study and one with obvious ethical implications, participants at the death dinner events were invited and had some knowledge of my initial research in the area. Insitutional Review Board ethical approval was secured, and participants were not compensated for their participation. The data collected was not shared beyond research dissemination and this was agreed to when consent was obtained. Recordings of the dinners are housed within the PaR submission on the Figshare repository owned by the University of Salford.

The ages of the participants ranged from early thirties to late sixties, with an equal gender balance. In all, 15 participants took part, 5 at each meal. It was not possible to include participants from a range of socioeconomic backgrounds or health experiences and this is something that needs attention going forward. Every dinner included one person working in the area of death and dying (e.g. a death doula, a celebrant and a palliative care nurse). This professional perspective was important as it provided a lynchpin for some of the practical conversations related to death and dying that developed over the course of the dinners.

Similarly, as the PaR submission developed, two participants of the dinners (from two different evenings) provided feedback on the analysis. The final PaR submission was also internally and externally reviewed as part of the process undertaken by the University of Salford for the UK Research Excellence Framework. Both of these endeavours were particularly helpful in ensuring the inter-rater reliability and triangulation of the data analysis and qualitative coding discussed later in the article.

### Procedure: The dinners themselves

Following a period of initial research, three death dinners were held. Aligning the events to a traditional three-act structure, each ‘course’/moment for discussion was punctuated with a live, performed monologue. This structure was deliberate as it allowed a pause for reflection on the themes within the monologue to occur naturally before those themes were discussed subsequently over in the next communal moment/‘course’. Similarly, the monologues were staged with a variety of dramaturgical and proxemic considerations, each taken to create the optimal environment to aid the participants’ comfort and connection with the material. As part of the PaR methodology, dramaturgical interventions in lighting and proxemics were thought out in detail and later reflected on and evaluated with the aid of the post-dinner questionnaires and video recordings.

The monologues were based on my own lived experience of death and explored personal concerns and considerations. The first explored my earliest experiences of death and dying and the impact these had on my subsequent understanding of and relationship to the subject. The second examined my feelings about death as an adult orphan and mother. The third and final monologue considered the notion of a ‘good’ death and what that would mean for me. At the end of each monologue, a question was posed to encourage reflection and conversation. There were:

1. Monologue 1: When did you first become aware of death and what impact did it have?
2. Monologue 2: What does death mean to you at this moment in time?
3. Monologue 3: Is there such a thing as a good death? What would that look like for you?

Engaging with the themes of each monologue in this way allowed a natural progression of the subject matter across the meal from the first to the last topic.

## Methodology and measures

The project adopted a mixed methodology that engaged PaR and qualitative research in the areas of death, dying, bereavement and palliative care as well as autobiographical performance, commensal practices and applied theatre. PaR is a practice-led methodology and critical framework that allows researchers to discover new knowledge and insight using creative methodologies, practices and outputs. Well established as a leading research methodology within the creative arts, PaR uses creative practice as a form of research that generates detectable research outputs.^12^

PaR rests on the premise that without the development of the practice and subsequent reflection upon that practice, the outcomes of the research cannot be known. Thus, in the case of the Death, Dinner, and Performance project, it was only through the act of creating the events themselves and experiencing them as the performer/host/researcher that the outcomes could be understood. As Smith and Dean argue, ‘creative practice – the training and specialised knowledge that creative practitioners have and the processes they engage in when they are making art – can lead to specialised research insights which can then be generalised and written up as research’^12^ (p. 5). In the Death, Dinner, and Performance project, the development of autobiographical monologues, as well as the dramaturgical arrangement of the events, comprise the specialist knowledge employed to create the theatrical prompt used to engage participants. To be answered, the research question required this specialist knowledge and ability to create, as well as the critical reflection undertaken after the dinners were held.

A PaR methodology is based on a being-thinking-doing module, which as Nelson explains, relies on ‘the attempt to *know-what*, to make the tacit more explicit’^13^ (p. 44). This, Nelson continues involves ‘the dynamic inter-relation between *know-how* and *know-that* to generate informed critical reflection’^13^ (p. 44). In the Death, Dinner, and Performance project, the ‘know how’ was gained through the experience of creating the event and the practice (autobiographical, commensal, dramaturgical) used within it, while the ‘know that’ came from the qualitative reading outlined above. The relationship between both, along with the critical reflection that occurred following the creative output, allows the ‘know what’ to be discovered. In this way, the implicit knowledge found through the creative practice was made explicit through critical reflection.

Qualitative data were also collected through pre- and post-questionnaires and video and audio recordings of the event. With an emphasis on being as unobtrusive as possible, participants were observed and recorded while dining. Four cameras in each corner of the room were placed at a distance of 5 m from the dinner table which was situated in the middle of the black box space. The dining table was lit overhead with a spotlight casting everything around the table in darkness, this added to the unobtrusiveness of the recording. The footage obtained was later examined in conjunction with the pre- and post-dinner questionnaires.

Participants were asked to fill in the pre-dinner questionnaire 1 week before the event answering the following questions:

1. Death is. . .
2. I think about death (circle as appropriate)? Often/Occasionally/Rarely/Never
3. I discuss death with others (circle as appropriate)?
4. Often Occasionally Rarely Never
5. The reason for this is. . .
6. When I think about death I feel. . .
7. I have considered how I would like my death to be (circle as appropriate). Yes/No
8. For me a good death would be . . .
9. Before I die I would like. . .

One week after the event, participants were sent the post-dinner questionnaire and asked to respond to the following questions:

1. Since the Death Dinner I have thought about death? More/Less/The same
2. Since the Death Dinner I have discussed death with others? More/Less/The same
3. The reason for this is?
4. Since the dinner I have reflected on how I would like my death to be. Yes/No
5. If yes, what would that be?
6. I have considered what I would need to put in place for this to happen. Yes/No
7. If yes, that is because?
8. If no, that is because?
9. Please comment on the experience of the death dinner and whether the structure/style of the event made you more or less likely to engage with the subject matter.

The data obtained from the questionnaires, combined with the recordings of the events, provided visual, aural and written records of responses to the prompts presented through the performance and staging/hosting of the death dinners.

Qualitative coding of participant responses allowed an inductive, thematic analysis which highlighted several recurring themes. These were analysed under two categories and are articulated in the Analysis and results section at the end of this article. The first category relates to the participants’ discussion around the subject of death and dying. The second category relates to the strategy (the commensality of the death dinners and the use of autobiographical performance) used to encourage that discussion. As well as data capture, pre- and post-questionnaires and video recording also allowed me to observe inter-rater reliability and triangulation between observations and self-reports. The PaR and qualitative methodology combined thus allowed me to explore the efficacy of the following:

1. Autobiographical performance and commensality to prompt conversation around death and dying.
2. Theatrical strategies to encourage those conversations to develop.
3. The death dinner event to encourage ongoing reflection in participants on death and dying and conversations therefrom.

## Discussion

### Autobiographical performance as a prompt

We cannot know what death is until it happens to us. Indeed, Heidegger^14^ notes that while most discussions on death (historical, biographical, ethnological, psychological, etc.) appear to come out of an inherent understanding of death, it is in fact only a presupposed concept of death that we ‘understand’. None of us really know what death is or how it will be experienced until it happens to us. Heidegger^14^ tries to punctuate the received wisdom that death is inevitable by suggesting that in fact, this thinking is a way of postponing death rather than recognising what is truly peculiar about it, namely, that it can happen at any and every moment. To talk about death then is to recognise its existence and conversely the existence of life. If death is inevitable and arbitrary so too is it integral to as May argues ‘a fullness of life that would not exist without it’^15^ (p. 4). While it is possible that Heidegger^14^ is correct, that death is the one thing that cannot be shared, our concerns about death are universal and exploring them may be something we can do together. As May continues, ‘thinking about death, leads us to think fruitfully about life’^15^ (p. 4).

One of the ways theatre allows us to engage with death is through the provision of a safe substitute, someone who acts for us, someone whose experiences of loss, death and bereavement we can experience at a distance. Considering this, while developing the Death, Dinner, and Performance project, I began to explore what Heddon calls the ‘here-and-nowness of autobiographical performance’ and the ‘visible presence of the performing subject – their here and nowness too’^16^ (p. 6). Rather than provide a fictional character through which an audience could experience death vicariously, I wanted to explore autobiographical performance to consider if personal experience might provide an inclusive prompt to encourage conversation.

In autobiographical performance, the mediation of experience is set apart from other modes.^16^ This, one could argue, is because of the intimate relationship that develops between performer and spectator in autobiographical performance. Although no less mediated than other forms of creative practice, the mediation that occurs in autobiographical performance has the potential to have an impact if capitalised on strategically.^16^ By positioning myself and my stories at the heart of the event, I was able to provide a level of distance from the subject matter for the participants. This meant participants could choose to discuss the subject personally or with a level of detachment, using my experiences to discuss the subject theoretically. Thus strategically, the autobiographical monologues and their mediation through the death dinners events provided both a prompt and a safety net, encouraging personal engagement when possible and ‘holding’ participants where necessary.

The monologues also provided a useful framing device with one participant noting they ‘were very effective in giving the event a structure and in bringing one into the evening’. Providing a chronological and thematic framework, they encouraged participants to consider their individual relationship to death and how this changed over the course of their lives and might evolve in the future. The monologues were therefore developed with an awareness of the unique temporality that exists in performance, what Heddon calls, ‘its here and nowness [. . .] its ability to respond to and engage with the present, while always keeping an eye on the future’^16^ (p. 2). This particular temporality worked to encourage engagement in a subject that, for all participants, has or will have an impact. However, it did so from a comfortable distance, allowing reflection and consideration; the level of response and engagement depended upon the amount of distance participants had at that moment from death, dying and bereavement.

While the monologues were written from a personal perspective, their accessible nature allowed universal themes to be explored communally. Out of the conversations prompted by the monologues came a number of themes. These included an acknowledgement of the importance of social and cultural response to death and dying; ritual and death and dying; types of deaths in relation to impact; and the taboo that surrounds violent or childhood deaths. These themes recurred in each of the dinners, their persistence highlighting the ability of the imbedded autobiographical material and adapted dramaturgy to facilitate the shift from personal to communal experience, and from there, onto a consideration of the subject more broadly in terms of society and culture.

The post-dinner questionnaire responses note how the monologues encouraged engagement and interaction with one participant stating, ‘the performances were thought provoking and sparked conversation’ and another offering, ‘the moments of the performance worked very well structurally to move the conversation into different areas, while also offering a hook for us to attach our responses to, to link back to and to reference in the course of the conversation’. Exposing my fears around death and dying in the monologues meant participants could remain in the conversation and talk about fear generally without having to expose their own. Using my experiences of bereavement, I could prompt a discussion and hold that discussion (and the participants) secure in the knowledge that we could be returning to those experiences if at any point the conversation became too upsetting for any one individual. As one participant reflected, ‘It was important to feel held, and to know that someone was leading the conversation [. . .]. It allowed us to relax and not feel responsible for anything other than thinking, reflecting, and sharing our experiences’.

### Theatrical strategies used in the death dinners

Commensality is defined as the practice of eating together. Within the Death, Dinner, and Performance project, commensality allowed the ontological nuance of participation to be explored. The root of commensality comes from the word mensa which means eating at the same table, a fundamentally social activity that both create and cement relationships.^17^ Some suggest commensality as an act is, in and of itself, an articulation of human society; its power being the fact that it does not rely on social and cultural homogeny.^18^ Indeed, as Simmell^19^ argues, ‘persons who in no way share any special interest can get together over a common meal [. . .] There lies the immeasurable social significance of the meal’^19^ (p. 130).

In contemporary performance practice, commensality, the act of coming together to commune over food, has long since been used (e.g. Reckless Sleepers’ The Last Supper, 2009 and Burtin’s The Midnight Soup, 2016). Socially engaged practice often uses commensality as a means to engage audience members/participants dialectically. In the Death, Dinner, and Performance project, the required communality and level of participation were high. Commensality worked to negate this. As one participant noted, ‘Having something else to do (i.e. eating) is always a really great way of conversation flowing in a more organic way than I think it does when the focus is entirely on having to make that conversation’. Indeed, almost all participants commented positively on the commensal element of the event. Their feedback included statements such as ‘Eating, drinking wine and talking about our demise at the same time was a comfort and a funny little contradiction’, ‘The experience for me was very heartening’. ‘I enjoyed talking and listening’ and ‘Eating and chatting with wine felt like an excellent formula!’.

Harpin and Nicholson argue that ‘participation promises authorship’^20^ (p. 10). That participation suggests through involvement, participants impact the ‘affective shape, atmosphere or political direction of the performance’^20^ (p. 15). However, performance that asks for participation is often highly controlled, the notion of co-authorship being more of a dramaturgical device than an attempt to create agency. Control was certainly a feature of the death dinners in terms of how the event was structured and how the discussion was prompted. Following that prompt in the form of the monologues; however, one could argue the discussions themselves resisted a hierarchical turn. No theatrical prowess or subject-specific expertise was required for the participant to have an autonomous ‘seat at the table’ so to speak. Their engagement was not sought to entertain others or to move the action of the piece forward. Responses were not pre-empted, nor did they not have a defined role within the piece. Each course/monologue reflected a different personal/universal moment. As such, what followed could not ‘fail’ so there was no need to exhort control over the outcome. There is no ‘wrong’ response in the context of personal experience.

### Analysis and results

PaR reflection and analysis, along with qualitative coding of participant responses allowed an inductive, thematic analysis that highlighted several recurring themes. These are analysed and discussed here under two categories:

1. Recurring themes in the participants’ discussion around the subject of death and dying.
2. Recurring themes in relation to the socially engaged strategy (commensality and use of autobiographical performance) used to encourage that discussion.

### Recurring themes in the participants’ discussion

Over the course of the dinners, several themes emerged. Universally, there was a sense that the experience of death as a child impacts future thoughts and feelings around death and dying. Fear of dying, and particularly of having lived an unfulfilled life, was a recurring theme and one that appeared to correlate to a lack of personal experience of death and dying.

Types of death (e.g. sudden *versus* drawn out) were discussed and considered in relation to the ability to discuss one type of death more easily over another. So too was the relationship to the dead person after death. In one conversation, the notion of the person being sacred after death was deliberated with one participant coining the phrase ‘death draws a line under the truth of a person’.

Choice as to how one would like to die was considered, particularly in relation to debilitating illness and palliative care. So too was liminality and death; the journey to death as liminal, the home in long-term palliative care becoming a liminal space and the liminal position loved ones inhabit while caring for a person on a palliative journey. Interestingly, one participant expressed having mixed feelings about death, trepidation and excitement, comparing their feeling to those associated with creativity and artistic practice stating, ‘death feels precipitous and visceral. It is like being on the verge of an imaginary death – that is, doing something creatively, physically, and/or emotionally charged and sublime’.

A number of participants in the dinners who confessed to thinking about death regularly but not discussing their thoughts openly with loved ones in the pre-dinner questionnaires shared a wish to do so following the experience of the event. The pre-dinner questionnaire showed eight participants thought about death occasionally, while seven stated they rarely spoke with others about death. This is interesting when compared with the post-dinner responses which showed four participants had thought more about death since the dinner and five had discussed death more with others following the event. Similarly, several participants who stated they had not previously considered putting in place plans regarding their death, commented this was now something they would/had discussed with loved ones. One participant went so far as to state the dinner had spurred them on to complete a will.

All participants involved commented that the experience of the death dinner was a positive one as can be seen in the following example, ‘The experience was very rich, I think. Overall, despite thinking that I wouldn’t (or wouldn’t be able) to engage with discussions on the topic of death, I felt that the event and experience really opened up a space where I could contribute, share, and learn from others’ experiences too. Thank you’.

While those who work in death-related areas did not claim to have discussed death more as a result of the experience, other participants, not regularly exposed to the subject provided clear anecdotal evidence of engaging with it more as a result of attending one of the dinners. For example, one participant stated, ‘The death dinner discussions, by their nature, led to some reflection after the event about some specific experiences of death, as well as wider thoughts about how I engage with death on a day-to-day basis. I discussed these thoughts with my partner, and we reflected on how we, as a couple, discuss and engage with death’.

Several participants expressed a wish to take action to ensure their wishes regarding their death and dying were known by loved ones. These included making a will (traditional and living) talking directly to loved ones about their wishes and revising/amending plans as needed. Those who did not express such a wish explored why they felt that reluctance which was enlightening in and of itself, for example, ‘I had the opportunity to discuss this with my mum last week and didn’t raise the topic (so I’m clearly still reluctant to discuss it in certain situations)’.

### Recurring themes in relation to the socially engaged strategy

Within the post-dinner questionnaires, attendees of the death dinners were asked to consider the performance elements used in the event and the impact these had on their experience. In relation to the autobiographical performance and its ability to prompt conversations on death and dying, participant responses showed the following: the importance of silence and proxemics, the positive experience of being hosted and the ability of autobiography to provide both a prompt and a safe haven. Dramaturgically, the importance of pace, timing and allowing space for contemplation amongst the participants also became evident from responses.

Also of relevance was the importance of silence and the realisation that different types of silence occur in relation to different emotional states. Recognising when moments of silence occurred was interesting in and of itself. Noting these moments and what it was about the conversation at that particular point that created either the silence of an individual, or the silence of the group, allowed subsequent reflection and a consideration of different types of silence (i.e. emotional, awkward, contemplative) to occur.

In most participatory performances, the silence of the participants might be read as negative and suggest a lack of engagement or interest. However, in the death dinners, silence was often read positively, as a sign that participants were contemplating the themes to discuss them more openly. Once this was recognised, the silences happened and were embraced and rarely, if ever, felt uncomfortable or inappropriate.

Proximity and the setting of the monologues at a distance from the conversation, as well as how participants entered and exited the space, all impacted their engagement with the material and the understanding of the event as framed by these devices. Reflection on these elements between the events allowed changes to be made and later analysed. For example, reflection on the movement of participants from the event back into the ‘real’ world encouraged the introduction of a ‘cool down’ exercise to aid the transition.

Most participants commented positively on the experience of being hosted. They also remarked on the structure of the event and its positive impact on their ability to engage fully with the subject, for example, ‘The dinner made the engagement seem informal and provided a suitable forum for discussion about something so fundamental’. Participants also acknowledged the autobiographical material as a helpful prompt. For example, one noted, ‘I haven’t thought in too much detail yet, but hearing you speak about how you would like your death to be, did really make me sit up and take notice’. Also responding to my prompt regarding a good death, another participant noted,

I suppose it’s quite similar to what was described in the performance. I hope to be old and have lived a full life. To have my family and some close friends nearby and to die peacefully without too much pain or long-drawn-out illness. To have photos and music around me and to remember what a full and beautiful life I’ve lived.

When commenting on the performance helping them to discuss death further, participants reacted positively particularly in relation to the impacts of the monologues and performative moments embedded within the event. For example, one participant noted, ‘The ‘readings’ between courses really helped to remind us of the issues around death and stimulated the discussions. They stimulated the imagination, and it reminded me in some ways of a Burns Supper – formal for the poetry and speech elements; ritualistic like everything that surrounds notions of dying and death; and human in the social contact and comfort afforded by the dining together’.

## Strengths and limitations

Limitations and considerations for future directions include a wish to engage a more diverse set of participants with different health experiences and socioeconomic backgrounds. Longevity in terms of impact could also be further explored with a set of questionnaires taken at two later dates (e.g. 1 month/6 months after the event).

With the responses above in mind, the project is now entering another period of research and development where the PaR methodology will be further employed. The newly titled Can We Talk About Death? project will consider how the practice can be developed to include the voices of the death dinner participants and the themes articulated over the course of the dinners. Through a process of adaptation, the interaction will move from an intimate, commensal encounter to a public-facing performance with a communal audience. This change in relationality will allow the impact of that adaptation to be examined as well as the changes to dramaturgy and performance practice that will be needed to facilitate that adaptation.

Removing the commensality and changing the performance strategy from intimate to communal will allow me to consider the outcome when the creative frame is altered. What will be especially interesting is how this shift impacts the position of autobiographical performance as a prompt and how the relationship between performer/participant changes when a certain level of relationality and intimacy is lost.

## Conclusion

This article has outlined the Death, Dinner, and Performance project and its use of commensality and an autobiographical performance strategy to encourage conversation around the difficult subject of death and dying. It did so while articulating the findings of the project, both in terms of the participant’s responses to the performative prompts provided and what was learned in terms of the practice itself and its impact.

The level of engagement in the dinners and responses from participants following the events show a positive impact on participant attitudes towards death and dying going forward. These included tangible actions on behalf of participants to have their death wishes known as well as prompting conversations between participants and their loved ones. Responses show that even participants reluctant to have these conversations had begun to consider why that is and what they could do going forward to allow those conversations to develop.

Ultimately, the project created new insight and knowledge into the role of autobiographical performance to prompt, frame and engage people in difficult discussions around death, dying and grief. And, while the questionnaires have proved useful in understanding the participants’ responses, the knowledge gained from the dinner themselves, from developing the strategy that allowed them to occur and from hosting the events, has been invaluable.

Findings, both from participant data and the PaR methodology, will now be used to develop the project further. The outcomes of the Can We Talk About Death? project remain to be seen but can only be uncovered through the PaR being-thinking-doing module outlined at the beginning of this article.

## Declarations

**Ethics approval and consent to participate:** Informed consent was obtained from all participants; IRB: Arts and Media Research Ethics Panel, University of Salford; Ethical approval numbers/IDs (AMR1718-15); participants were not compensated for their participation. The data collected were not shared beyond research dissemination and this was agreed to when consent was obtained. Recordings of the dinners are housed within the Practice as Research submission on the Figshare repository owned by the University of Salford.

**Consent for publication:** Please note that this section refers to patient/participant/guardian consent for publication (e.g. of patient images/data reported in a case report or case series), not author consent for publication. I confirm that I have obtained written informed consent to publish this article.

**Availability of data and materials:** Figshare link: http://usir.salford.ac.uk/id/eprint/50890/
